# Ossifying renal tumor of infancy: a case report

**DOI:** 10.3389/fonc.2023.1301328

**Published:** 2023-12-12

**Authors:** Yu Qu, Guoqiang Du, Feng Guo, Rongde Wu, Wei Liu

**Affiliations:** Department of Pediatric Surgery, Shandong Provincial Hospital Affiliated to Shandong First Medical University, Jinan, China

**Keywords:** ossifying renal tumor of infancy, diagnosis, pathology, prognosis, partial nephrectomy

## Abstract

Ossifying renal tumor of infancy (ORTI) is an extremely rare benign renal solid tumor with typical clinical and pathological features. Most cases are diagnosed in infants that are less than 12 months of age and is more common in males. The first symptom in most patients is painless gross hematuria. Microscopically, the tumor has three main components: an osteoid core, osteoblast-like cells, and spindle cells. We reported a case of a 21-day-old patient diagnosed with ORTI who underwent partial nephrectomy and had good follow-up. The unique features of this case are the strong expression of Wilms Tumor-1 (WT-1) and a high Ki-67 index in the hot spot area. ORTI is considered to have a favorable prognosis. Due to the rarity of WT-1 positivity and high Ki-67 index, we should be highly aware that this patient needs to be followed closely. In addition, we reviewed the available literature on ORTI, with the aim of summarizing the diagnostic and therapeutic experience. The diagnosis needs to be given cautiously on the basis of clinical symptoms, imaging, and pathologic examination. Depending on the location and extent of the tumor, surgery can be performed by partial nephrectomy or nephrectomy to avoid overtreatment.

## 1 Introduction

Ossifying renal tumor of infancy (ORTI) is an extremely rare benign renal solid tumor. Most cases are diagnosed in infants that are less than 12 months of age and is more common in males (1). By reviewing the English literature on PubMed, only 25 cases of ORTI have been reported to date since first reported by Chatten and colleagues in 1980 (2). Patients tend to present to the hospital with painless gross hematuria, rarely as an abdominal mass. This typical clinical feature and imaging data may aid in the diagnosis, and pathologic information is key for making a definitive diagnosis. This tumor is a benign lesion, and almost all reported patients have remained well postoperatively, without recurrence or metastasis. ORTI should be distinguished from other renal solid tumors in clinical practice. In this study, we report a case of ORTI and review the literature, with the aim of summarizing its diagnostic experience, improving the diagnostic rate, and avoiding unnecessary treatment.

## 2 Case presentation

**Figure 1:**
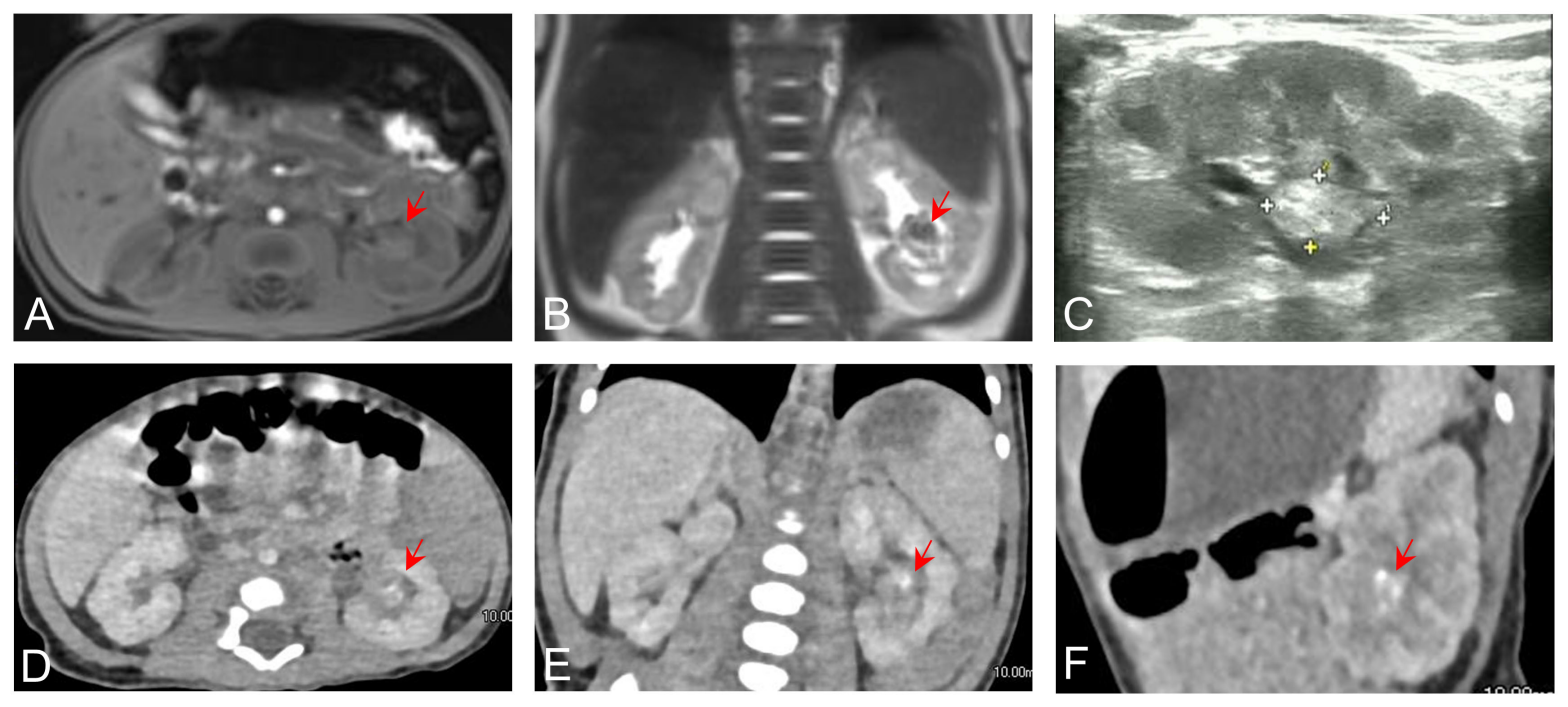
Axial **(A)** and coronal **(B)** preoperative MRI images showing abnormal signal foci in the renal pelvic region. **(C)** Ultrasound showing calcification in the left renal pelvis. Axial **(D)**, coronal **(E)**, and sagittal **(F)** contrast-enhanced CT images showing abnormal enhancement in the left renal pelvis.

**Figure 2:**
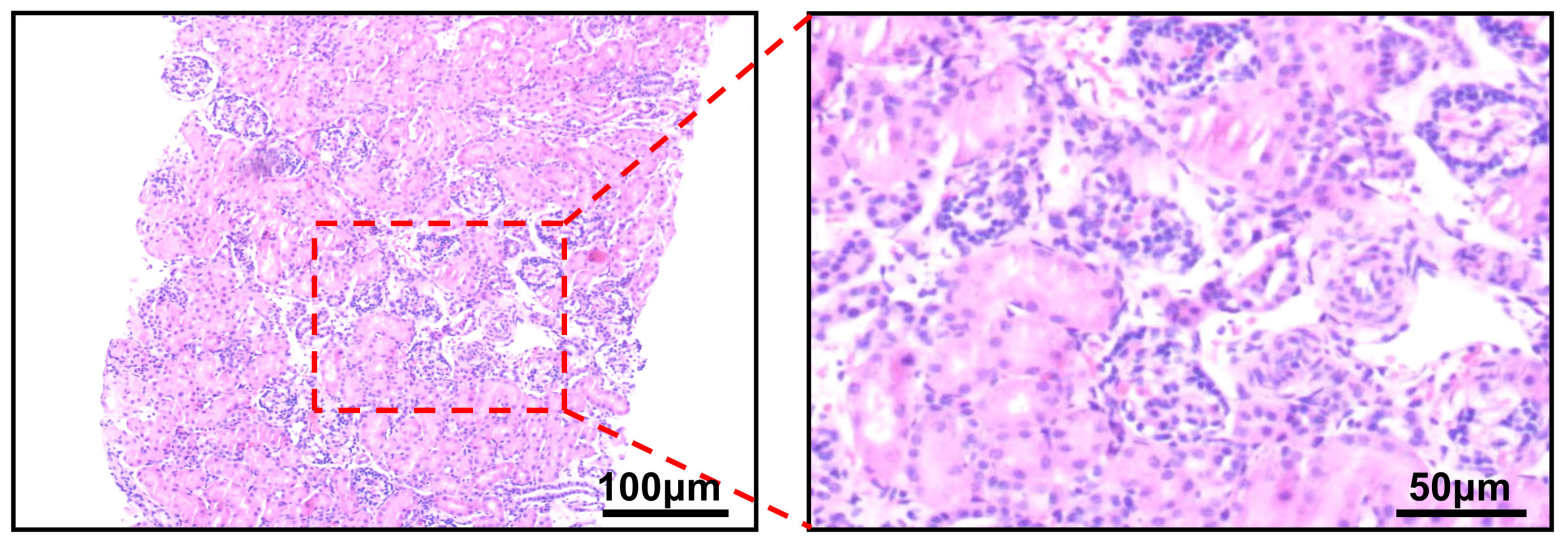
Puncture pathology showing the presence of small patches of spindle-shaped cells with interstitial vitreous degeneration and no mitotic image.

A 21-day-old infant was found to have painless gross hematuria lasting 8 h after birth, which did not improve after symptomatic treatment, such as anti-inflammatory and hemostatic treatment. Routine urinalysis revealed an erythrocyte count up to 2,691 per microliter and 349.8 per high power field. Urologic ultrasound showed a hypoechoic mass in the middle and lower part of the left renal collecting system, measuring approximately 2.2×1.1×1.1 cm (**Figure 1C**). Enhanced computed tomography (CT) was then performed, which showed abnormal enhancement with localized calcification in the left renal pelvis (**Figures 1D–F**). Magnetic resonance imaging (MRI) results showed abnormal signal foci in the left renal pelvic region (**Figures 1A, B**). All of these signs provided evidence to suspect a neoplastic lesion. Therefore, ultrasound-guided renal puncture was performed. The puncture was performed by specialized staff of the department of interventional ultrasound, under ultrasound monitoring throughout the entire process. After local anesthesia, two strips of tissue from the left renal mass were punctured with a type 18G biopsy needle under ultrasound guidance. The exfoliated cells from the punctured tissue were detected and the remaining tissue was sent for pathological examination. The patient had no signs of bleeding on ultrasound and was given a local dressing. Exfoliative cytology showed that one cell type was seen, which was scattered and distributed in small clusters, with a consistent cytosol size, rounded and off-set nuclei, 2–3 nucleoli, and abundant cytoplasm, considered as tumor cells. Puncture pathology showed small patches of spindle cells with no mitotic image, favoring a benign lesion (**Figure 2**).

**Figure 3:**
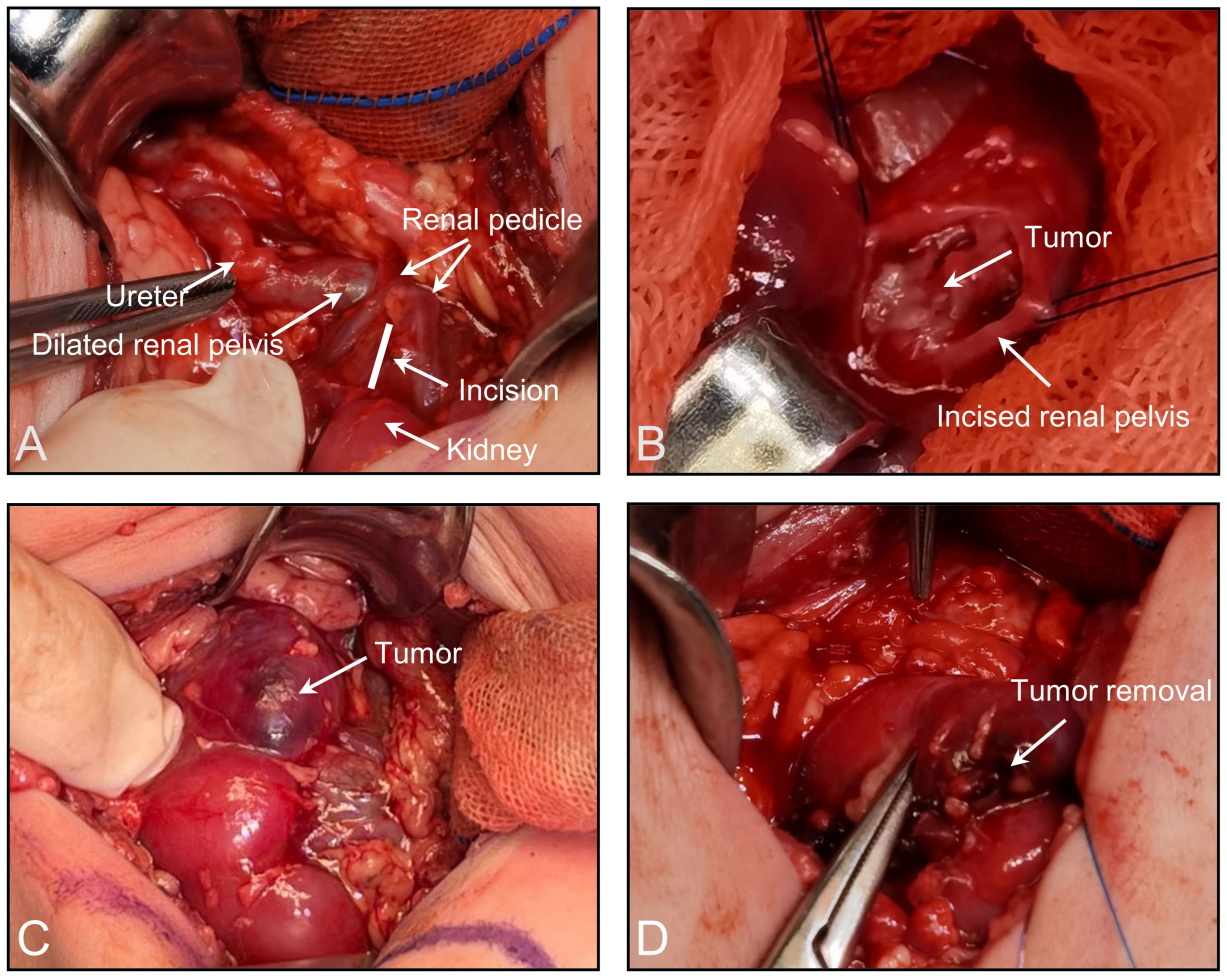
**(A)** The renal pelvis was incised transversely between two branch vessels along the middle and lower pole of the left kidney. **(B)** A cauliflower-like lump was seen. **(C)** Partially collapsed lower pole of the left kidney. **(D)** Stripping and removal of the mass after incising the renal cortex.

After several diagnostic procedures, the renal pelvic mass was resected and a final histologic diagnosis of ORTI was made. Intraoperatively, the renal pelvis was significantly dilated, with a tortuous ureteropelvic junction, and a slight narrowing of the local ureter. Freeing the renal clitoral vessels on the surface of the renal pelvis, the renal pelvis was incised transversely between two branch vessels along the middle and lower pole of the left kidney (**Figure 3A**). A cauliflower-like lump was seen (**Figure 3B**). Upon exploration, the tumor was seen to emanate from the left lower renal calyx. The tumor was enucleated along the border of the tumor by approximately 5 mm to ensure a negative tumor margin. A localized collapse at the lower pole of the kidney was seen; the renal cortex was incised from there and the residual mass was stripped and removed (**Figures 3C, D**). The renal cortex and renal pelvis were sutured, and the renal pelvic outflow tract was checked for patency. The intraoperative frozen pathology result was ORTI. Consequently, the affected kidney was preserved. A double J-tube, model F3, with a length of 120 mm, was placed inside the ureter.

**Figure 4:**
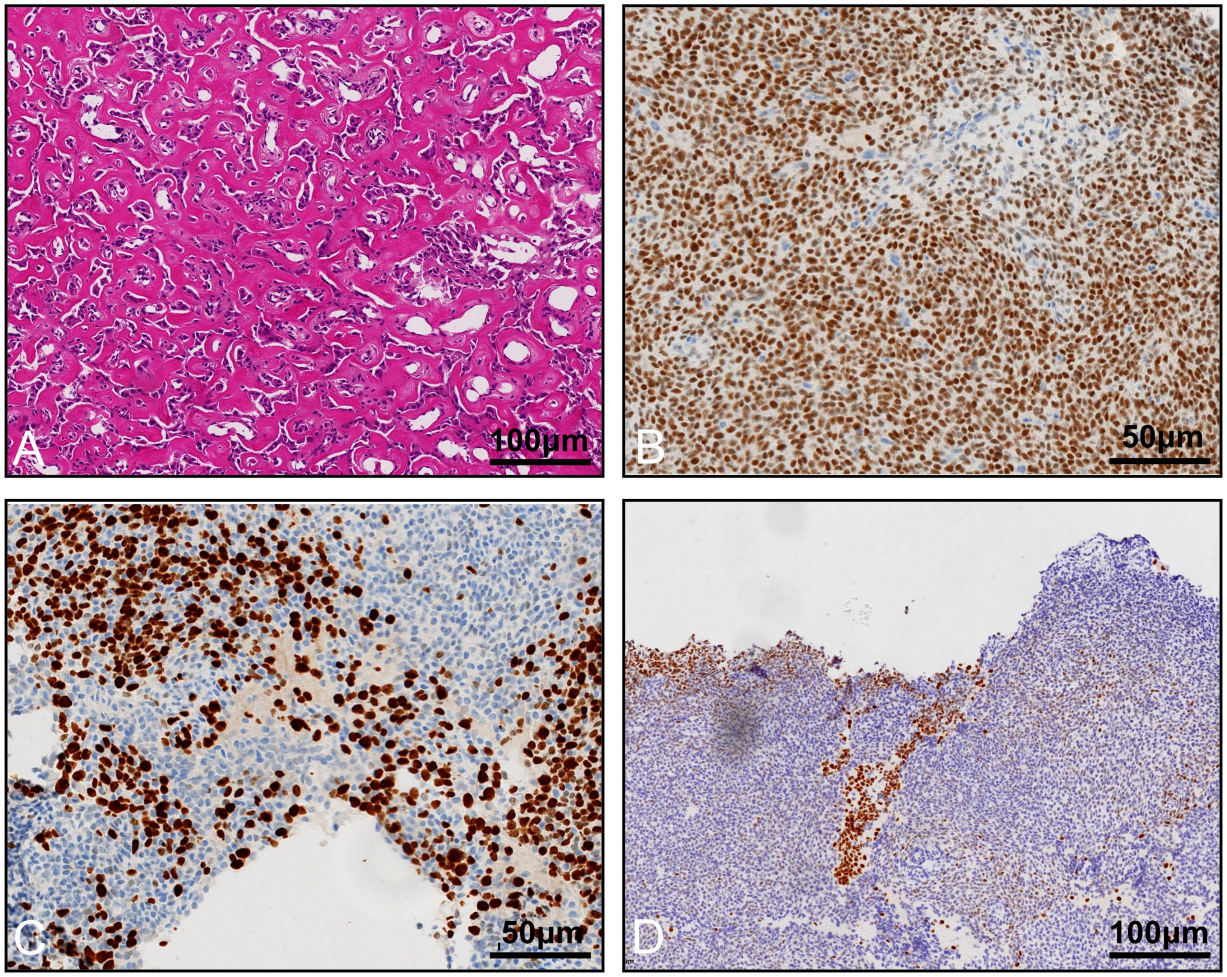
Histological appearance and immunohistochemistry staining of the tumor. **(A)** Osteoblast-like cells in an osteoid core, HE staining. Immunohistochemistry staining reveals that the tumor cells are positive for WT-1 **(B)**, Ki-67 **(C)**, and STAB2 **(D)**.

The final pathology revealed ossifying renal tumor of infancy (**Figure 4**). Immunohistochemical results showed the following: Vimentin (+), Wilms Tumor-1(WT-1) (+), Cyclin D1 (+, partial), CK (-), EMA (-), INI-1 (+), Brg1 (+), Actin (-), Desmin (-), PAX8 (-), CD34 (-), S100 (-), Myogenin (-), MyoD1 (-), and STAT6 (-). Moreover, osteoblast-like cells were positive for STAB2. Approximately 60% of the cells were positive for Ki-67. The patient only had partial nephrectomy, and no other radiation or chemotherapy was given. The patient underwent urologic ultrasound, with no significant findings during postoperative retaining of the double J-tube. The double J-tube was removed at 68 days postoperatively. Up to now, the child has been followed up for 9 months, and no tumor recurrence or metastasis has been detected. The latest re-examined ultrasound results showed that the left kidney size was 5.7×3.7×3.2 cm, renal parenchyma thickness was 1.1 cm, and the collecting system was slightly separated and approximately 0.6 cm wide.

## 3 Discussion

ORTI is an extremely rare type of renal tumor in infants and young children; it is a benign tumor with a good prognosis. There are only 25 published cases in the literature. There are no statistics on the prevalence or incidence of ORTI in single centers or regions. In the previously reported cases, the age at diagnosis ranged from 6 days to 30 months, and most were diagnosed before the first year of life. It predominantly occurred in males. The most common clinical symptom was gross hematuria, and only two of the previous cases presented with a palpable abdominal mass on exam as the main symptom (1). On imaging, most cases showed a mass in the renal pelvis and calyces with clear borders, often with calcification (1). It is often accompanied by calcification and shows no or moderate enhancement on CT. Calcification may not be evident because of the patient’s young age or the small diameter of the tumor (3). Low T2 signal in MRI is a characteristic of this tumor (3, 4).

Microscopically, the tumor has three main components: an osteoid core, osteoblast-like cells, and spindle cells. The central part of the tumor was an osteoid core, which is the typical morphological feature of this tumor, and the proportion and maturity of the osteoid core increased with age (5). Focal osteoblast-like cells were seen between the osteoid core, which were polygonal with large nuclei and abundant cytoplasm. The osteoid core was surrounded by spindle cells with ovoid or spindle-shaped nuclei, fine chromatin, and sparse cytoplasm. The immunohistochemical staining of spindle cells was positive for Vimentin and WT-1, positive for SMA, and negative for EMA and CK in the literature. The osteoblast-like cells were positive for Vimentin, SATB2, EMA, and CK. Both cellular components were positive for Vimentin and INI1 and did not express CD99, Desmin, Myogenin, MYOD1, or CD34. The immunohistochemical results in this case fully support the diagnosis of ORTI.

At present, the histologic origin of ORTI is not well defined. The earliest researchers recognized that it originated from the urethral epithelium (2). Some studies have suggested that ORTI is a subtype of congenital mesangial nephroma (CMN) (6). It has also been suggested that the transformation from spindle cells to osteoblast-like cells suggests that the osteoblast-like cells are derived from intralobular nephrogenic rests (ILNR). Some authors hypothesize that ORTI may arise from hyperplastic ILNR because of the similarity between nephrogenic rests (NR) and the spindle cells in ORTI, and that Wilms tumor (WT) and ORTI may share a common pathogenic pathway (7). Some scholars believed that that the tumor may be a stage in the process of WT formation that tends toward benign differentiation (8). ORTI should also be differentiated from several diseases. Tumors can be polypoid or staghorn-like, extending into the collecting system, even leading to hydronephrosis or dilatation of the renal pelvis and calyces (9). It can be misdiagnosed as a staghorn stone on CT. CMN is common in infants below 6 months of age, and microscopically, consists mainly of a spindle cell component similar to that found in ORTI. The absence of an osteoid core and osteoblast-like cellular components in the tumor, as well as the detection of the ETV6-NTRK3 fusion gene, can differentiate from ORTI. NR have mesenchymal tissue; however, no bone-like tissue or osteoblast-like cells are present to differentiate them. WT is the most common malignant tumor of the kidney. The average age of a child with nephroblastoma is approximately 3 years old. Histology consisted of an undifferentiated embryo, with epithelial and mesenchymal components. Immunohistochemistry of WT showed positive WT-1, yet ORTI is uncommon. Vaillancourt and colleagues reported the first case of an ossifying renal tumor of infancy, positive for WT-1 immunohistochemistry staining, in 2017 (10). In addition, this patient also showed a high expression of Ki-67, which implied that tumor cells are more active in proliferation and are more aggressive. Whether the detection of this feature affects the prognosis of the patient needs to be verified over a longer follow-up period. In recent years, the chromosomal karyotype of ORTI has been reported in the literature with clonal trisomy 4 (11), which is considered to be characteristic of this tumor and differentiates it from other renal tumors in infants and children.

Partial nephrectomy or nephrectomy was seen as the main treatment modality in previously reported cases, and chemotherapy in the minority. Previous cases were well followed up, and no cases of metastasis and relapse have been detected yet, which shows the favorable prognosis for patients with ORTI. One of the patients with the longest follow-up of 23 years showed no progression (12), confirming its benign nature. When the tumor is of appropriate size and location, nephron sparing surgery can provide adequate treatment outcomes and protect as much renal function as possible. Considering that the pathologic findings in this patient showed a low probability of invasiveness, we recommended that the patient undergo urologic ultrasound every 3 months postoperatively and enhanced CT of the urinary system at 1 year postoperatively.

## 4 Conclusion

We reported a case of ORTI positive for WT-1 and with a high Ki-67 index. ORTI is extremely rare in clinical practice, diagnosed by its typical clinical symptoms and specific microscopic features. Surgery can be performed by partial nephrectomy or nephrectomy, depending on the location and extent of the tumor, and the prognosis is favorable. Due to the rarity of WT-1 positivity and the high Ki-67 index in this case, regular follow-up is required.

## Data availability statement

The original contributions presented in the study are included in the article/supplementary material. Further inquiries can be directed to the corresponding author.

## Ethics statement

The studies involving humans were approved by Biomedical Research Ethic Committee of Shandong Provincial Hospital. The studies were conducted in accordance with the local legislation and institutional requirements. Written informed consent for participation in this study was provided by the participants’ legal guardians/next of kin. Written informed consent was obtained from the individual(s), and minor(s)’ legal guardian/next of kin, for the publication of any potentially identifiable images or data included in this article.

## Author contributions

YQ: Conceptualization, Data curation, Formal Analysis, Investigation, Methodology, Project administration, Resources, Software, Validation, Visualization, Writing – original draft, Writing – review & editing. GD: Conceptualization, Data curation, Formal Analysis, Investigation, Methodology, Project administration, Resources, Software, Validation, Visualization, Writing – original draft, Writing – review & editing. FG: Conceptualization, Data curation, Formal Analysis, Investigation, Methodology, Project administration, Resources, Software, Validation, Writing – original draft, Writing – review & editing. RW: Formal Analysis, Funding acquisition, Investigation, Methodology, Project administration, Resources, Software, Supervision, Validation, Visualization, Writing – original draft, Writing – review & editing. WL: Formal Analysis, Funding acquisition, Investigation, Methodology, Project administration, Resources, Software, Supervision, Validation, Visualization, Writing – original draft, Writing – review & editing.
